# Safety and Immunogenicity of the Live Attenuated Vaccine QazCOVID-Live Against Coronavirus Infection COVID-19: Pre-Clinical Study Results

**DOI:** 10.3390/vaccines12121401

**Published:** 2024-12-12

**Authors:** Lespek Kutumbetov, Balzhan Myrzakhmetova, Aiganym Tussipova, Gulzhan Zhapparova, Talshyngul Tlenchiyeva, Karina Bissenbayeva, Kuanysh Zhapar, Kuandyk Zhugunissov, Sergazy Nurabayev, Aslan Kerimbayev

**Affiliations:** Research Institute for Biological Safety Problems, Gvardeiskiy 080409, Kazakhstan; lespek.k@gmail.com (L.K.); aiganym.t24@gmail.com (A.T.); gulzhan1003@mail.ru (G.Z.); t.m.tlenchieva@mail.ru (T.T.); bisenbayeva.karina@bk.ru (K.B.); zhapar.kzk@gmail.com (K.Z.); kuandyk_83@mail.ru (K.Z.); sergazy-75@mail.ru (S.N.); aslan_kerim@mail.ru (A.K.)

**Keywords:** QazCOVID-live, live attenuated vaccine, SARS-CoV-2, COVID-19 prevention, preclinical study, vaccine safety, virus attenuation, virus-neutralizing antibodies

## Abstract

The research conducted in this preclinical study assesses QazCovid-live, a live attenuated COVID-19 vaccine created in Kazakhstan, by conducting preclinical evaluations of safety, immunogenicity, and allergenicity in various animal models, including mice, rats, hamsters, and guinea pigs. The vaccine, developed by attenuating SARS-CoV-2 via numerous Vero cell passages, had no significant adverse effects in acute and subacute toxicity assessments, even at elevated dosages. Allergenicity testing indicated the absence of both immediate and delayed hypersensitivity reactions. Immunogenicity evaluations revealed strong virus-neutralizing antibody responses, especially following intranasal and intratracheal delivery. Studies on reversibility and transmission further validated the vaccine’s stability and non-pathogenicity. The data indicate that QazCovid-live is safe, immunogenic, and prepared for clinical trials, presenting a potential strategy for COVID-19 prevention.

## 1. Introduction

The global outbreak of SARS-CoV-2, the virus that causes COVID-19, has produced an unprecedented public health problem, with millions of deaths and considerable economic impact [1,2,3]. COVID-19 affected the world from 2020 to 2022, with over 580 million confirmed cases and 6.4 million deaths [4]. Safe and effective vaccines are urgently needed to contain the pandemic. Focus on rapid vaccination platforms such as mRNA, adenoviral vectors, DNA vectors, inactivated and subunit vaccines for COVID-19 has led to the rapid release of COVID-19 vaccines for human use. These first-generation vaccines represent significant progress [1].

However, not all known vaccines can totally beat SARS-CoV-2 due to their limited efficacy and performance, with the exception of the underappreciated live attenuated vaccines (LAV), which can have significantly higher efficacy and performance compared to other vaccines [5,6,7]. LAVs have eliminated far more harmful viruses than any other vaccines in history. The production of live attenuated vaccines adheres to a well-established vaccinology principle: weaken a virus to minimize its pathogenicity while retaining its capacity to elicit a significant immune response [8,9]. For decades, live attenuated vaccinations have been used successfully to prevent infections like measles [10], mumps [11], and rubella [12], providing long-term protection by triggering both humoral and cellular immune responses [13].

LAVs can entirely prevent infection and transmission of viruses and their variations. As a result, they can prevent the establishment of vaccine-resistant and virulent variations, as well as better protect immunologically abnormal individuals [14,15,16,17,18]. Animal tests and clinical trials can ensure the safety of LAV against COVID-19. A small number of registered LAVs have demonstrated safety in animal models and are currently undergoing clinical studies [8,9,16,19,20,21].

Among several vaccine research efforts throughout the world, Kazakhstan has created its own live attenuated vaccine, QazCovid-live, led by the Research Institute for Biosafety Problems (RIBSP).

This preclinical study is aimed to assess the safety, immunogenicity, and allergenicity of the QazCovid-live vaccination in a variety of animal species, including BALB/c mice, rats, Syrian hamsters, and guinea pigs. The outcomes of this research provide vital data to support the vaccine’s safety and efficacy, setting the groundwork for moving the vaccine into clinical trials.

## 2. Materials and Methods

### 2.1. Ethical Approval

Research was executed in compliance with ethical regulations and standards: Guidelines of the Council of Europe Convention for the Protection of Vertebrate Animals used for Experimental and Other Scientific Purposes; Directive from the Council of the EAC, suggestions from the FELASA Working Group Report (1994–1996) [22,23].

### 2.2. Vaccine

#### 2.2.1. Virus Attenuation and Cultivation

Vero cell cultures were employed to conduct the attenuation process for the SARS-CoV-2 virus. The susceptibility of eleven unique primary and continuous cell lines to SARS-CoV-2 infection was assessed. Vero cells were chosen for viral propagation following an evaluation of sensitivity and viral replication efficiency. Viral titers and the rate of cytopathic effect (CPE) manifestation were all assessed.

To reduce pathogenicity while maintaining immunogenicity, the virus underwent many passages. The cytopathogenic effects reached stability following five passages. After 24 h, at specific infection multiplicities, cytopathic effects (CPE) became apparent, and, subsequently, between 72 and 96 h, the entire cell monolayer degenerated. At every assessed passage level, the characteristics of the pathogen’s CPE remained consistent and exhibited dynamic changes: cellular swelling and rounding, creation of intercellular spaces, and the desquamation of cells from the adhesion surface individually. Within 24 to 72 h, 80 to 100% of cells exhibited this cytopathology. To guarantee population homogeneity, the virus was cloned at passages 71–72, 81–82, and 91–92 using the limited dilution method. The cloning process did not influence the cytopathogenic activity of the virus’s progeny populations.

#### 2.2.2. Virus Purification

After the culture was harvested, the viral suspension was purified using tangential flow ultrafiltration to remove cellular debris and contaminants. The virus was further purified through exclusion chromatography, which helped concentrate the virus and separate it from unwanted proteins or cellular material. The purification process involved filtering the viral suspension using 0.22-micron membrane filters (Sigma Aldrich, Saint Louis, MO, USA) to ensure sterility.

During the filtration stage, the clogging of filters was a recurring issue. This was resolved by incorporating centrifugation at 3000 rpm for 30 min, which significantly reduced debris and allowed for smoother filtration. The filtration process was improved, cutting down the purification time from 5 days to 24 h.

#### 2.2.3. Lyophilization (Freeze-Drying)

To stabilize the virus for long-term storage, the purified viral suspension was mixed with a protectant solution containing lactalbumin hydrolysate, sucrose, and sorbitol (Sigma Aldrich, Saint Louis, MO, USA). The mixture was then freeze-dried using the lyophilization technique. This process allowed the virus to retain its activity while being stored in a dried form for future use.

#### 2.2.4. Genetic Sequencing and Cloning of the Virus

The genetic analysis of the virus was conducted at various passages to ensure its stability and uniformity. The sequencing of the S gene was conducted with the Sanger dideoxy method on an AB3130xl (Hitachi Applied Biosystems, Foster City, CA, USA) 16-capillary genetic analyzer autosequencer, employing the BigDye Terminator 3.1 cycle sequencing kit (ABI, Foster City, CA, USA) and specifically designed primers. Genetic sequencing of the spike protein gene (S gene) was performed at passages 33 and 100. For uniformity, viral clones were produced at the 71–72, 81–82, and 91–92 passages using the limiting dilution method. This cloning process did not affect the cytopathogenic activity of the virus.

### 2.3. Preclinical Safety Studies

#### 2.3.1. Acute Toxicity

An acute toxicity study was performed to assess the immediate effects of a high dosage of QazCovid-live. The research used rats (190–210 g, 10–16 weeks old) and BALB/c mice (20–22 g, 6–8 weeks old) of both sexes, including groups of pregnant specimens. The animals were arbitrarily assigned to control and experimental groups. The vaccine was administered intramuscularly to the experimental groups at the maximum allowable volume: five injections at one-hour intervals, equating to one human dosage (0.5 mL) for mice and ten human doses (5 mL) for rats. The control group animals received saline in an equivalent volume. The dosages were determined in accordance with the standards for the experimental (preclinical) investigation of novel pharmacological agents [24,25].

Daily clinical observations of the animals were conducted for 14 days post-vaccination. Monitored criteria encompassed: overall health, including dietary and hydration intake; state of integument, pelage, and mucosal membranes; behavioral responses and locomotor activity.

Body weight was measured at baseline (before vaccination), 24 h, 7 days, and 14 days after vaccination. Following a 14-day period, the animals were euthanized via decapitation, and a visual examination of the internal organs was conducted.

#### 2.3.2. Subacute Toxicity

**Figure 1:**
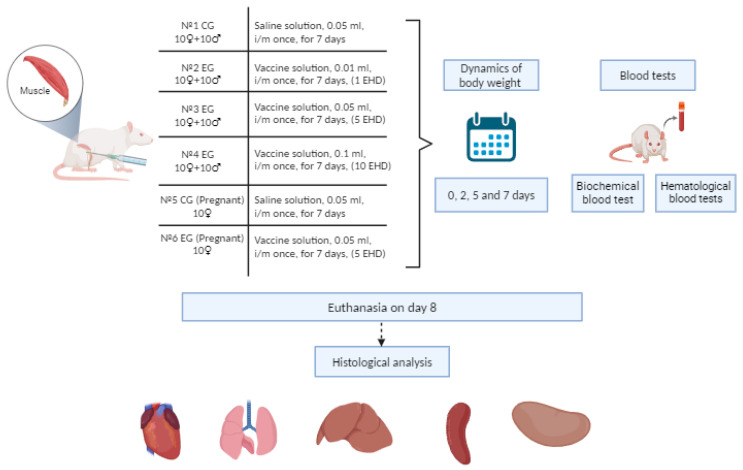
Flowchart of the process in the subacute toxicity investigation. Created with BioRender.com.

QazCovid-live was provided many times over a seven-day period as part of subacute toxicity evaluations. The study utilized rats weighing between 180 and 220 g, encompassing both sexes. Four groups of rats were used: one as a control (PBS) and three for experimental purposes. The attributes of the examined rat groups are depicted in Figure 1. The evaluation of the subacute (subchronic) toxicity of the vaccine was conducted by delivering three doses to the animals. The selected doses were determined by the findings of initial acute toxicity tests, in consideration of the anticipated maximum therapeutic dose advised for clinical application. The vaccine was delivered intramuscularly once daily for seven days, adhering to the GLP RK standard and the Guidelines for Experimental (Preclinical) Study of New Pharmacological Substances [26]. The control group animals received saline in an equivalent volume via the same administration route for a duration of 7 days. Daily clinical observations were conducted, emphasizing weight variations, overall health, and behavioral activity.

Upon completion of the trial, the animals were euthanized, and their internal organs—liver, kidneys, and lungs—were analyzed for pathological alterations. The investigation also encompassed a histological analysis to evaluate potential tissue injury.

#### 2.3.3. Residual Pathogenicity of the Attenuated SARS-CoV-2 Virus

To assess the residual pathogenicity and infectivity of the attenuated SARS-CoV-2 virus at the 80th and 100th passages, groups of five Syrian hamsters were inoculated using different routes: intranasal, intratracheal, subcutaneous, and intravenous. The viral dose used was 10^4.5^ TCID_50_ per animal. A control group was also infected intranasally with a virulent strain of the virus at the 5th passage. Pathogenicity was assessed based on clinical signs, disease severity, and the presence of pathologies in the animals.

#### 2.3.4. Safety Assessment in Mice

To evaluate the safety of the attenuated virus, outbred white BALB/c mice were administered the virus intraperitoneally at a dose of 0.5 cm^3^. The mice were monitored daily for clinical symptoms, and their body weight was recorded to detect any negative health impacts.

#### 2.3.5. Reversibility and Transmission Studies

The reversibility of the attenuated virus was tested on Syrian hamsters, ferrets, and kittens via intranasal inoculation. Clinical symptoms, weight gain, pathological examinations, and histological studies of lung tissues were used to assess virus behavior. Virus transmission was evaluated by co-housing vaccinated animals with uninfected animals to assess whether the attenuated virus could be transmitted and cause disease in previously uninfected animals.

#### 2.3.6. Immunogenicity and Safety Testing of Different Administration Routes

The immunogenic activity of the vaccine was assessed on Syrian hamsters inoculated via four routes: intranasal, intratracheal, subcutaneous, and intramuscular. A single dose of 0.2 mL/animal containing 10^6.0^ TCID_50_ of the attenuated virus was administered. Blood serum samples were collected on days 21 and 28 post-vaccination to measure virus-neutralizing antibody (VNA) titers. Safety was also assessed by monitoring the general health and weight gain of the animals.

#### 2.3.7. Comparative Immunogenicity of Different Doses

Syrian hamsters were immunized via intramuscular and intranasal routes using virus doses of 10^2.0^, 10^3.0^, 10^5.0^, and 10^7.0^ TCID_50/animal_. VNA titers were measured on days 14, 21, 28, 35, and 42 post-vaccination. The safety of the virus was assessed through daily clinical evaluations and body weight monitoring.

### 2.4. Allergenicity Study

The investigation into the allergenic characteristics of the QazCOVID-live vaccination was performed on guinea pigs and white inbred mice. The experiment comprised healthy individuals of both genders. Variation in the beginning weight did not surpass 10%.

The allergenic effect of the vaccine was assessed using a general anaphylactic reaction test, a skin application method, delayed-type hypersensitivity, and a conjunctival test.

#### 2.4.1. Immediate-Type Hypersensitivity (Anaphylaxis)

Immediate-type hypersensitivity (ITH) (anaphylaxis) is characterized by the identification of allergic reactions mediated by IgE, IgG, and IgM, which may manifest within minutes or hours following exposure. Animals were categorized into control and experimental groups, with each group receiving either the research medication at varying effective therapeutic doses (ETD) or a saline solution. The W.O. Weigle index was computed based on the intensity of allergic reactions, encompassing fatalities, severe shock, moderate shock, and mild reactions.

#### 2.4.2. Delayed-Type Hypersensitivity

Delayed-type hypersensitivity (DTH) results from the interaction between the antigen (allergen) and macrophages, together with Th1 cells, which activates cellular immunity that manifests 1–3 days post-exposure. Mice were administered the vaccination and sensitized using sheep erythrocytes.

The sensitizing injection was provided once via the intraperitoneal route, while the resolving injection was administered once into the pad of the hind paw. Ram erythrocytes served as a positive control material. The sensitizing dose of the positive control vaccine was 2 × 10^8^ cells, while the resolving dose was 2 × 10^9^ cells. A physiological solution served as the negative control vaccine. The duration of observation was six days.

The experimental medication (vaccine) and physiological solution (control) were administered one day prior to sensitization, concurrently with sensitization, and 24 h post-sensitization using ram erythrocytes in a volume of 0.02 mL. Sensitization was conducted with a single intraperitoneal injection of ram erythrocytes at a concentration of 2 × 10^8^ cells in a volume of 100 μL. On the fifth day of the experiment, a resolving dose of ram erythrocytes (2 × 10^9^) in a volume of 50 μL was administered into the pad of the right hind paw of all animal groups, while 50 μL of sterile physiological solution was injected into the left hind paw. The reaction outcomes were documented after 24 h.

Upon conclusion of the experiment, the animals were beheaded, after which both paws were severed above the heel joint and subsequently weighed. The impact of the medication on the progression of delayed-type hypersensitivity (DTH) was evaluated by the paw edema index in both experimental and control groups for each mouse separately. The disparity in paw edema between the experimental and control groups was utilized to compute the edema index (EI).

The edema index is calculated using the formula:

EI = [(Mo − Mk)/Mk] × 100%, where

EI—is the edema index (%);

Mo—is the weight of the foot of the paw into the pad of which the ram erythrocytes were injected;

Mk—is the weight of the foot of the paw into the pad of which the physiological solution was injected.

#### 2.4.3. Skin Application Test

The investigation of the sensitizing impact through skin application methods was conducted on 10 albino guinea pigs, each weighing between 300 and 330 g, comprising 5 in the experimental group and 5 in the control group.

The test vaccination was administered to the depilated lateral surface of the animals, about at the midsection, with 3–5 drops equally distributed over the entire area using an eye glass pipette daily for two weeks, resulting in a total of 10 skin applications. The control group animals were administered a saline solution. The responses were evaluated on a validated cutaneous reaction scale.

#### 2.4.4. Conjunctival Test Results

To conduct a conjunctival test, 2–3 drops of the vaccination solution were administered behind the upper eyelid of the guinea pigs utilizing a pipette with an extended narrow tip. The control group animals received an injection of 2–3 drops of physiological solution. Ocular instillation was conducted in a supine position, with the head oriented downward. The reaction was evaluated after 15 min and subsequently after 24–48 h utilizing a redness and irritation assessment scale.

#### 2.4.5. Skin Irritation Study in Rats

The skin irritating impact was evaluated on rats weighing 200 g. An amount of 0.5 mL of the vaccine was injected intramuscularly into the posterior quadriceps muscle of the thigh. Three days post-vaccination, the animals were decapitated, and skin tissue samples measuring 15 × 15 mm were collected from the injection site. The skin samples were rinsed in a physiological solution and subsequently immersed in a 10% formalin solution for additional histological analysis. The vaccine’s irritating effect was evaluated based on the histological analysis of the skin’s structure.

### 2.5. Statistical Analysis

Statistical analysis of preclinical animal group data was conducted using two-way repeated measures ANOVA, followed by multiple comparisons using the Gisser–Greenhouse correction test. Comparisons of protective efficacy among animal groups were conducted using the one-way Fisher’s exact test, with *p* < 0.05 being significant and means reported with standard errors (SEM). Statistical analysis of all experimental data was conducted utilizing Graph Pad Prism software version 8.0 (Graph Pad Software Inc., La Jolla, CA, USA). The experiments were replicated, yielding consistent findings. The GMT analysis utilizing Dunnett’s multiple comparison test with a 95% confidence interval following the first and second immunizations indicated statistically significant differences between the experimental and control groups administered commercial PBS (** = *p* < 0.0002, *** = *p* < 0.001).

## 3. Results

### 3.1. Vaccine

#### 3.1.1. Virus Attenuation and Cultivation

In Vero cell cultures, the SARS-CoV-2 virus showed stable cytopathogenic effects after five passages. Within 24 h post-inoculation, the virus induced cytopathic effects at certain multiplicities of infection, and within 72 to 96 h, the entire cell monolayer deteriorated. At all evaluated passage levels, the characteristics of the pathogen’s cytopathic effect (CPE) remained consistent and exhibited the following dynamics: cellular swelling and rounding, creation of intercellular spaces, and desquamation of cells from the adhesion surface individually.

Table 1 presents the outcomes of virus passages, indicating that the cytopathogenicity of the virus remained consistent across all passage levels. The viral titer at the 33rd passage and beyond varied from 10^6.33^ to 10^7.67^ TCID_50/mL_, contingent upon the infection dose.

#### 3.1.2. Purification

The data in Table 2 indicates that the initial two purification batches utilizing culture virus suspensions of 18 and 31 L, with titers of 10^7.00^ and 10^6.50^ TCID_50/0.1_, respectively, produced 1.0 and 1.6 L of purified virus concentrate with titers of 10^5.50^ and 10^5.25^ TCID_50/0.1_, respectively. The overall yield of purified virus relative to the initial quantity was 0.17% and 0.29%.

The quantitative titration results indicated that, following the clarification process via centrifugation, the overall yield of the purified virus amounted to 5.88% of the initial virus present in the culture suspension. This quantity was 20 times or more greater than the quantity of virus obtained without centrifugation. The qualitative parameters of purified virus samples conformed to the standards necessary for raw materials used in immunobiological preparations, such as vaccines. Experimental batches of the vaccine were prepared using the purified virus samples following stabilization and freeze-drying in vials.

#### 3.1.3. Lyophilization

The purified virus was dried via sublimation in the presence of stabilizing components, including lactalbumin hydrolysate, sucrose, lactose, and sorbitol. This process preserved the structural integrity and biological activity of the virus. The parameters of the dry virus following sublimation dehydration are detailed in Table 3.

As can be seen from the data in Table 3, the results of the standardization of the quality of the dry preparation indicate that the optimal formulation of the protective solution comprises lactalbumin hydrolyzate at a final concentration of 2.5%, sucrose at a final concentration of 4.0%, and injection water. The incorporation of sorbitol into the protective medium adds complexity to the dehydration process, leading to an extended drying duration and a notable decrease in virus titer, resulting in a product that fails to achieve a compact form. The dehydration mode significantly influences the shelf life of the dried virus. The findings indicate that a final drying period of 6 h leads to a significant decrease in the virus’s reproductive activity (by 1.25 lg). Conversely, shortening this period to 4 h allows for a notable preservation of its biological activity. The drying mode, which includes a final drying period of 4 h, shows a reduction in the virus titer of no more than 0.25–0.5 lg.

The stabilizing compounds effectively preserved viral activity over an extended period. Optimal storage conditions for the dried virus were determined to be temperatures below 8 °C, under which the virus retained its biological activity for at least 180 days (the duration of the observation period).

These findings highlight the critical role of scientifically validated stabilizing agents in the vaccine formulation, ensuring the drug’s efficacy and significantly extending its shelf life across various storage conditions.

All batches of the final vaccine passed the quality control tests, meeting the required standards for sterility, endotoxin levels, and absence of cellular DNA. This ensured the safety of the vaccine for experimental and potential clinical use.

#### 3.1.4. Genetic Stability

Genetic sequencing revealed two stable nucleotide substitutions in the S gene at positions 21,249 and 21,864, which remained present from the 33rd to the 100th passage. These mutations were responsible for the attenuation of the virus while maintaining its immunogenic properties. No other significant genetic changes were observed in the virus during these passages, indicating the virus’s genetic stability.

The attenuated virus strain was deposited in the RSE Microorganism Collection of the Research Institute for Biological Safety Problems, and a patent was obtained for the strain in Kazakhstan (Patent No. 35175).

### 3.2. Preclinical Safety Studies

#### 3.2.1. Acute Toxicity

Animal mortality following vaccination is one of the most important markers of acute toxicity. No deaths were reported in this trial in either the experimental or control groups, even those who received the highest dose of QazCovid-live that is permitted. As a result, the vaccination’s LD_50_ could not be determined.

**Figure 2:**
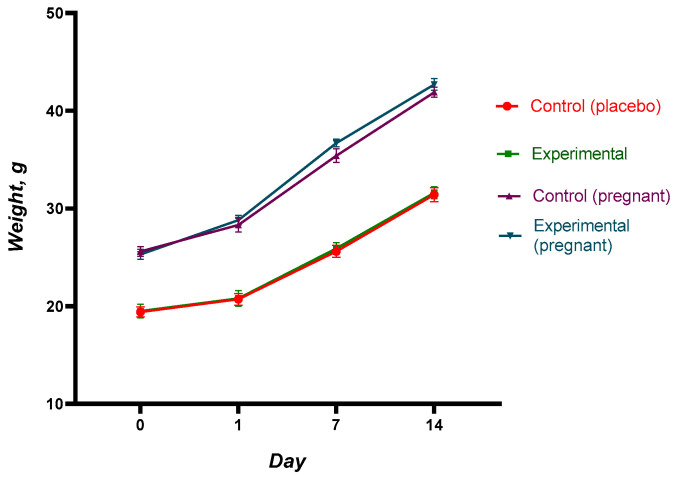
Dynamics of body weight of animals in the acute toxicity experiment (BALB/c mice). Changes in body weight were monitored in BALB/c mice over a 14-day observation period following the administration of QazCovid-live vaccine at dose equivalent to 1× the human therapeutic dose. All groups, including the control group, exhibited a stable increase in body weight. No statistically significant differences were observed between the experimental and control groups, confirming that the vaccine does not affect metabolism or growth. Each value represents the mean ± SEM of all animals in each group.

**Figure 3:**
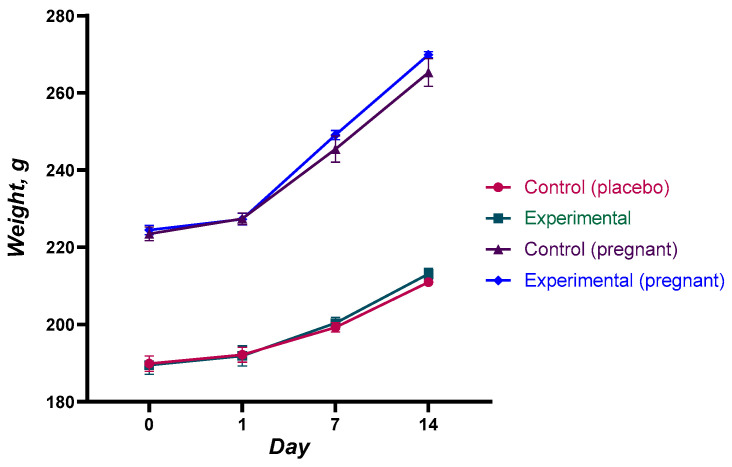
Dynamics of body weight of animals in the acute toxicity experiment (rats). Changes in body weight were monitored in rats over a 14-day period following the administration of the QazCovid-live vaccine at dose equivalent to 10× the human therapeutic dose. Positive body weight dynamics were observed in all groups, including both pregnant and non-pregnant animals. No significant differences were detected between the experimental and control groups, further confirming the safety of the vaccine. Each value represents the mean ±SEM of all animals in each group.

Body weight, feed and water consumption, the state of the skin, fur, and mucosa, as well as the animals’ behavioral responses and motor activity, were all closely observed during the trial. There was no discernible difference in the animals’ body weight between the experimental and control groups. Throughout the course of the investigation, the body weight of every animal showed a consistent increase. This shows that the immunization did not negatively effect the general metabolism or growth of the animals. The specific weight gain information is displayed in Figure 2 and Figure 3.

Throughout the observation period, no abnormal vital signs were seen in the experimental groups. The animals’ skin was smooth and their fur was lustrous and even. They looked healthy. Additionally, they reacted appropriately to visual, aural, and tactile stimuli. The rate of body weight rise in the experimental groups on days 6–7 following immunization was equivalent to that of the control group animals. These findings are noteworthy because they show that there is no adverse effect of the QazCovid-live vaccination on body weight or overall health.

The acute toxicity trial showed that the maximal dose of the vaccine (0.5 mL for mice and 50 mL for rats) was tolerated by both sexes of rats and mice, including pregnant animals, with no discernible differences from the control group. Throughout the investigation, the animals’ general health, the kind and degree of their motor activity, their motor coordination, the tone of their skeletal muscles, and the color of their mucous membranes all stayed constant. The animals’ tail position and other respiratory characteristics, such as breathing frequency and depth, were the same as those in the control group.

**Figure 4:**
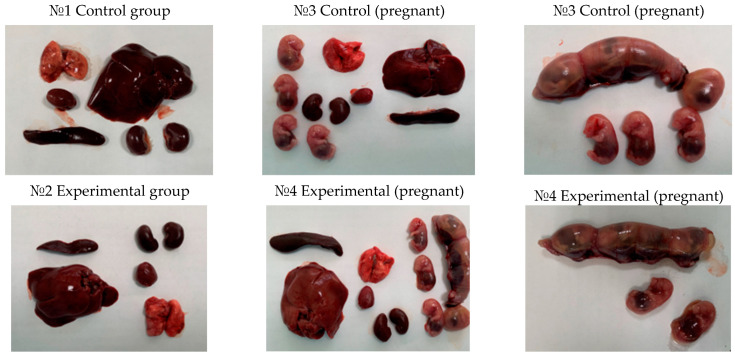
Macroscopic examination of internal organs of rats. Following autopsy, a macroscopic examination of the internal organs, including the liver, heart, lungs, kidneys, and spleen, was conducted in rats from both the control and experimental groups. No pathological changes, such as inflammation, necrosis, or structural abnormalities, were observed.

**Figure 5:**
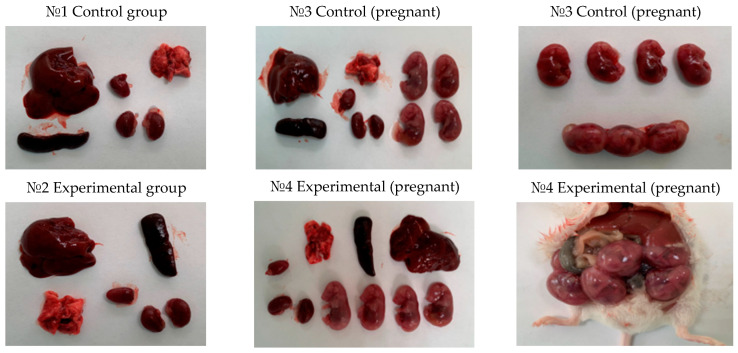
Macroscopic examination of internal organs of mice. Following autopsy, a macroscopic examination of the internal organs, including the liver, heart, lungs, kidneys, and spleen, was conducted in mice from both the control and experimental groups. In all groups, the organs exhibited a normal appearance, with no signs of inflammation, necrosis, or other abnormalities, confirming the vaccine’s tolerability.

The experiment ended with the animals being put to sleep and necropsies being done. There were no appreciable variations between the experimental and control groups when the internal organs were examined under a microscope. The liver, heart, lungs, kidneys, and spleen were among the organs that were visually normal; there were no indications of inflammation, necrosis, or other pathological abnormalities. Figure 4 and Figure 5, which show the macroscopic outcomes from the experimental and control groups of rats and mice, respectively, provide an illustration of these findings.

To sum up, the acute toxicity study verified that mice and rats, including pregnant ones, tolerate the QazCovid-live vaccination well and that there are no harmful consequences or abnormalities in normal physiological conditions.

#### 3.2.2. Subacute Toxicity

The effects of several (7-day) intramuscular doses of the QazCovid-live vaccination on laboratory rats were assessed in the subacute toxicity research. The investigation included both male and female animals, as well as pregnant females, and attempted to examine body weight changes, general health status, and behavioral reactions, in addition to potential biochemical, hematological, and histological changes in the internal organs.

All experimental groups, including those receiving the greatest dosages (up to 10 times the therapeutic human dose equivalent), did not experience any fatality during the course of the study. The safety of the vaccine at these doses is confirmed by the 0% mortality that was seen in all groups, including pregnant animals.

**Figure 6:**
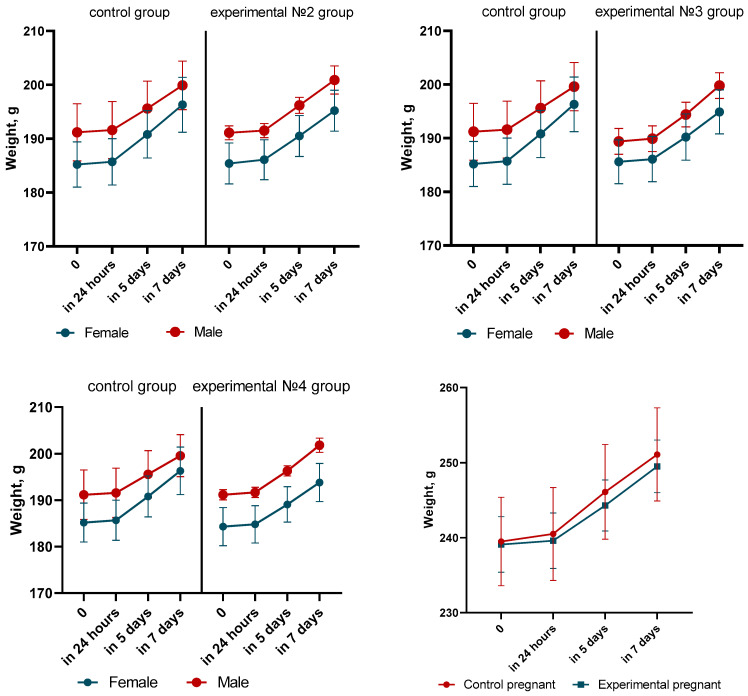
Dynamics of body weight of experimental rats. Body weight changes were monitored in rats following the administration of the QazCovid-live vaccine at doses equivalent to 1×, 5×, and 10× the human therapeutic dose. All animals exhibited stable body weight growth throughout the experiment. No significant differences were observed between the experimental and control groups, indicating that the vaccine did not adversely affect growth or metabolism. Each value represents the mean ± SEM of all animals in each group.

During the whole observation period, the experimental animals’ body weight dynamics were positive. All animal species, male and female, including pregnant females, showed a constant increase in body weight. No matter the dose level (1×, 5×, or 10× the equivalent human dose), the results, as indicated in Figure 6, show that there were no statistically significant changes in weight growth between the control and experimental groups.

Daily observations revealed that the animals’ overall health was unaffected by the vaccination, even at greater dosages. In terms of motor activity, behavioral reactions to stimuli (including tactile, auditory, and visual stimuli), or the state of the fur and mucosa, there were no discernible variations between the experimental and control groups.

There were no indications of motor dysfunction or problems with coordination; the intensity and kind of movement stayed constant.

The internal organs of the experimental animals showed no appreciable topographical or anatomical alterations during necropsy. The macroscopic examination revealed that the lungs, heart, liver, spleen, and kidneys of the vaccinated animals appeared healthy, without evidence of inflammation, necrosis, or structural abnormalities. Visually, the vaccinated groups’ organs resembled those of the control group. Table 4 displays the precise organ mass coefficients, which indicate that the experimental animals’ relative organ weights fell within normal ranges and did not exhibit a significant difference from the control group.

**Figure 7:**
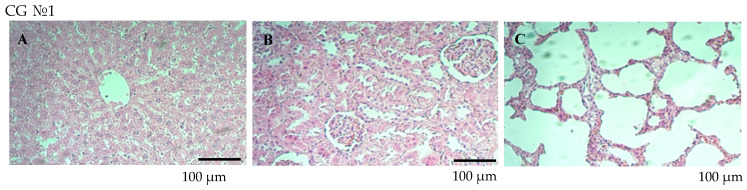

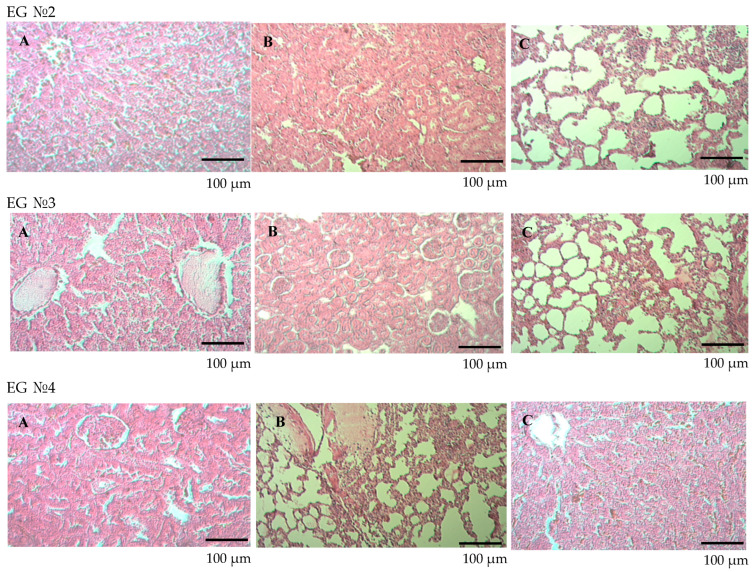
Histological analysis of different organs in the control and experimental groups that received different doses of the QazCovid-live vaccine. Histological sections of the liver (**A**), kidney (**B**), and lung (**C**) tissues were prepared and stained with hematoxylin and eosin (HE × 200) to assess tissue structure and potential pathological changes. The experimental groups received the QazCovid-live vaccine in doses corresponding to 1-fold, 5-fold, and 10-fold the therapeutic dose for humans, administered intramuscularly. The control group was administered a saline solution. The results demonstrate favorable tolerability of the vaccine at standard and moderate doses. High doses may induce localized changes in tissues, particularly in the liver and lungs; however, these effects remain within acceptable physiological limits. Scale bar: 100 µm.

**Figure 8:**
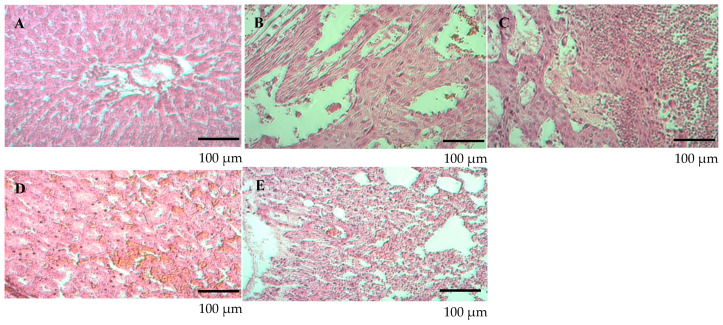
Histological analysis of various organs in Experimental Group 5. Histological sections of the liver (**A**–**C**), kidney (**D**), and lung (**E**) tissues were prepared and stained with hematoxylin and eosin (HE × 200) to evaluate tissue structure and potential pathological changes. Experimental Group 5 received the QazCovid-live vaccine at a dose equivalent to five times the therapeutic dose for humans, administered intramuscularly.

The parenchymal cells and stroma of the internal organs, such as the liver, kidneys, and lungs, did not exhibit any indications of dystrophic, destructive, or sclerotic alterations. There were slight indications of stromal edema in the liver and kidneys of the highest dose group (10× human dose equivalent), but no appreciable pathological alterations were found. Due to septal cell growth, a modest thickening of the interalveolar septa was seen in the lungs. These alterations were slight and did not point to significant tissue injury. Histological scans (Figure 7 and Figure 8) showed that the liver tissue in all groups had a normal lobular shape with central veins and radially arranged hepatocytes.

As shown in Figure 7, the control group exhibited a normal histological structure across all tissues, with no signs of edema, degeneration, or inflammation. In the 1× dose group (EG №2), the liver exhibited mild vascular congestion, minimal stromal edema was observed in the kidneys, and weak proliferation of septal cells, along with mild thickening of the interalveolar septa, was noted in the lungs. In the 5× dose group (EG №3), local edema without structural damage was observed in the liver, focal stromal edema was noted in the kidneys, and mild proliferation of septal cells, without significant disturbances, was observed in the lungs. In the 10× dose group (EG №4), foci of edema and parenchymatous degeneration of individual hepatocytes were detected in the liver. Focal stromal edema persisted in the kidneys, and moderate proliferation of septal cells, along with marked thickening of the interalveolar septa, was noted in the lungs.

As shown in Figure 8, the liver’s histological structure was generally intact; however, foci of edema and parenchymal degeneration of hepatocytes were observed. Signs of decidual hemorrhage and inflammation were detected in certain areas (Figure 8A–C). Focal hemorrhage and parenchymal degeneration of the tubular epithelium were observed in kidney tissue (Figure 8D). In the lungs, septal cell proliferation led to vascular congestion in the interalveolar septa (Figure 8E). These changes are associated with prolonged high-dose vaccine administration.

Biochemical Studies: After the animals had fasted for 14–15 h, blood samples were taken from them at the conclusion of the experiment. Analysis revealed altered levels of total protein, glucose, and ALT, with increased ALT observed in some groups compared to standard physiological ranges (Table 5). The decrease in total protein and glucose across all groups, including the control, was attributed to the 14–15 h fasting period prior to blood collection. Prolonged fasting depletes glycogen stores, leading to reduced blood glucose as the body shifts to gluconeogenesis. This effect is more pronounced in rodents due to their higher metabolic rate and limited glycogen reserves. The uniform decrease across all groups suggests that this is a normal physiological adaptation rather than a vaccine-related effect.

Serum protein levels are highly sensitive to food intake, and reduced dietary amino acid availability during fasting temporarily limits protein synthesis, contributing to the observed decrease in total protein levels. As this reduction was consistent across all groups, it is attributed to the fasting protocol rather than vaccine-induced toxicity.

The vaccine may have caused transient liver stress in some animals, leading to increased ALT release into circulation. This response may represent a normal immune or metabolic reaction to the administration of a live attenuated vaccine. The absence of changes in other biochemical markers of liver injury, such as bilirubin, alkaline phosphatase (ALP), or aspartate aminotransferase (AST), indicates that the observed increases in ALT are transient and not indicative of significant liver damage. Importantly, the lack of other markers of liver dysfunction suggests that these changes are not clinically significant.

Hematological parameters (Table 6), including total white blood cell count, neutrophil percentage, and markers of systemic inflammation (e.g., ESR), were within normal physiological limits. The increased lymphocyte counts observed in this study are attributable to species-specific characteristics of the rat immune system, which inherently exhibits a higher lymphocyte percentage (above 70%) compared to other mammals, including humans. Additionally, experimental conditions, such as fasting before blood collection, may have influenced these results.

The absence of significant differences between the control and experimental groups indicates that these changes are not associated with toxic or pathological effects of the vaccine. Furthermore, the increased lymphocyte counts in vaccinated animals reflect activation of the immune system, supporting the vaccine’s immunogenicity while confirming its safety.

The subacute toxicity study concluded that, even at dosages up to ten times the comparable human dose, QazCovid-live is well-tolerated in rats and does not appear to have any substantial harmful consequences. The vaccination has no negative effects on the animals’ internal organs or general health, as evidenced by the lack of mortality, normal weight gain, and stable biochemical and hematological markers.

#### 3.2.3. Residual Pathogenicity

Table 7 shows that all Syrian hamsters infected with the 80th and 100th passage viruses remained clinically healthy and alive throughout the observation period. No pathological findings were observed in the lungs of these animals. However, the control group, which was infected with the virulent strain at the 5th passage, exhibited clinical symptoms, weight loss, and hemorrhagic lesions in the lungs. This indicates that the attenuated virus from the 80th and 100th passages no longer has residual pathogenicity.

#### 3.2.4. Safety in BALB/c Mice

Mice inoculated with the attenuated virus showed no visible pathological effects, and both the experimental and control groups remained clinically healthy. Daily weight gain in both groups ranged between 0.8% and 1.0%, suggesting that the attenuated virus is safe for intraperitoneal administration in mice.

#### 3.2.5. Reversibility and Transmission

The results in Table 8 demonstrate that the attenuated virus was detected in the lung tissue of Syrian hamsters and ferrets but did not cause clinical disease or pathological changes. In kittens, no virus was detected in the lungs after three passages. The virus did not transmit from vaccinated animals to uninfected animals during co-housing, and no SARS-CoV-2 antibodies were found in the uninfected animals at the end of the study.

#### 3.2.6. Immunogenicity and Safety of Different Administration Routes

Syrian hamsters vaccinated intranasally and intratracheally with the attenuated virus developed the highest VNA titers (7–8 log_2_) by days 21 and 28 post-vaccination. Animals vaccinated intramuscularly and subcutaneously had lower VNA titers (3–5 log_2_ and 5–7 log_2_, respectively). No adverse clinical effects were observed in any of the animals, and body weight gain was consistent across all groups.

#### 3.2.7. Comparative Immunogenicity

**Figure 9:**
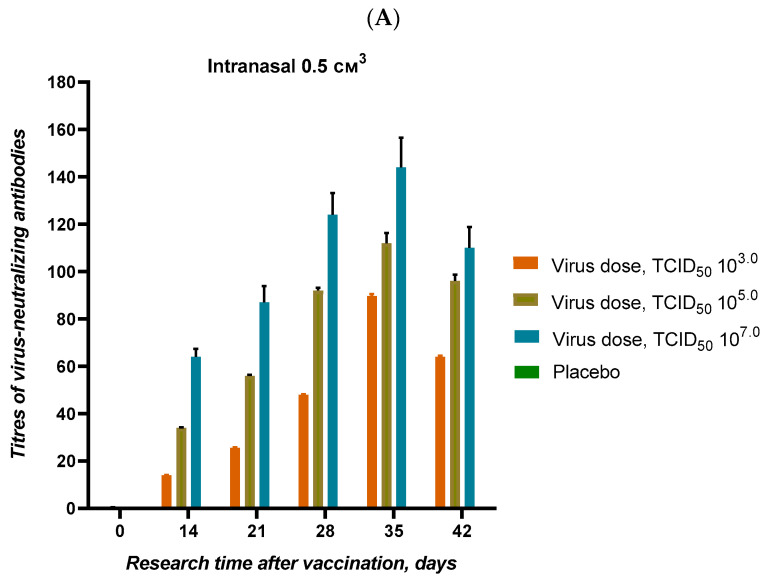

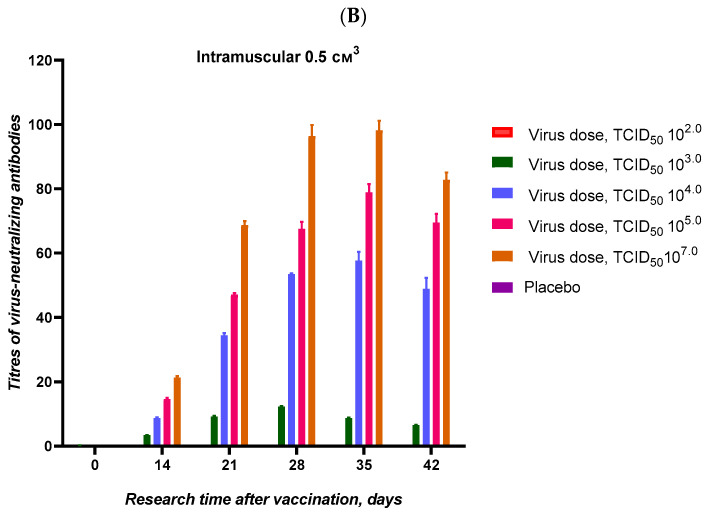
Titers of virus-neutralizing antibodies in the blood serum of Syrian hamsters vaccinated with the attenuated SARS-CoV-2 virus. Syrian hamsters were vaccinated with attenuated SARS-CoV-2 virus via intramuscular and intranasal routes at varying doses (1000 TCID_50_, 10^5.0^ TCID_50_, and 10^7.0^ TCID_50_). Serum virus-neutralizing antibody (VNA) titers were measured, and all doses elicited detectable VNA titers, with higher doses producing a stronger immune response. No pathological effects were observed. Each value represents the mean ± SEM of all animals in each group.

Immunogenicity studies revealed that a dose of 1000 TCID_50/animal_ was sufficient to induce detectable VNA titers in both intranasally and intramuscularly vaccinated animals (Figure 9A,B). Higher doses (10^5.0^ and 10^7.0^ TCID_50_) led to stronger immune responses, as indicated by higher VNA titers. The virus did not have any visible pathological effects on the animals, as evidenced by consistent weight gain across all groups.

### 3.3. Allergenicity Testing

#### 3.3.1. Immediate-Type Hypersensitivity (Anaphylaxis) Results

In the groups sensitized with the QazCovid-live vaccine, no anaphylactic reactions were observed. The W.O. Weigle index was calculated as zero for all experimental groups, indicating no signs of allergic shock across doses ranging from 0.01 mL to 0.3 mL.

#### 3.3.2. Delayed-Type Hypersensitivity Results

The vaccine did not induce significant delayed-type hypersensitivity reactions. The Edema Index (EI) in the experimental groups did not differ significantly from the control groups, as presented in Table 9.

No signs of swelling or inflammation were noted, suggesting that the vaccine did not cause allergic responses in this model.

#### 3.3.3. Skin Application Test

No erythema, edema, or other visible skin reactions were observed in any of the guinea pigs after 10 applications of the vaccine. The skin of the experimental animals was comparable to that of the control group. Table 10 summarizes the results.

#### 3.3.4. Conjunctival Test Results

There were no signs of conjunctival irritation or redness in either the experimental or control groups. Both immediate and delayed responses (after 24–48 h) were scored as zero across all animals.

#### 3.3.5. Skin Irritation Study in Rats

**Figure 10:**
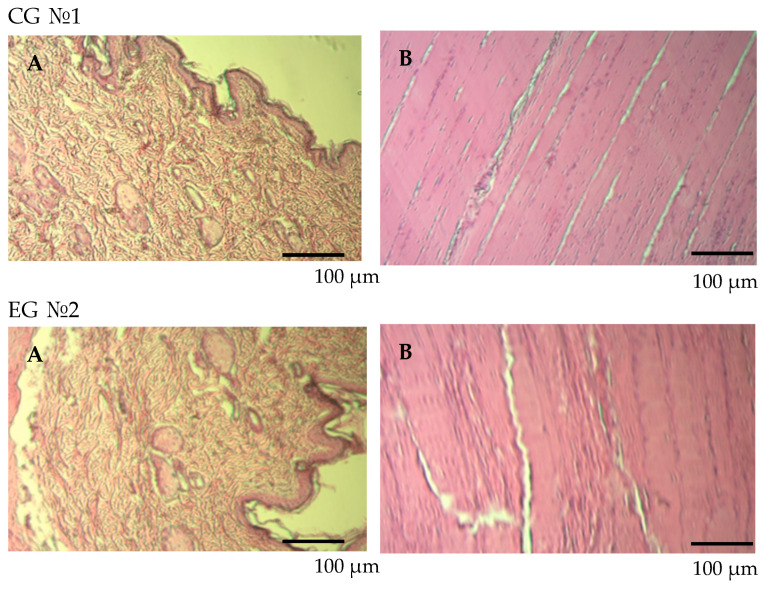
Histological analysis of skin and subcutaneous tissues at the vaccine injection site. Skin (**A**), muscle tissue (**B**). Rats were intramuscularly injected with 0.5 mL of the QazCovid-live vaccine into the posterior thigh. Histological sections from the injection site were stained with hematoxylin and eosin (HE × 100) to evaluate tissue structure. No pathological changes, including erythema, edema, necrosis, or irritation, were detected. Histological analysis confirmed preserved skin structure with minimal edema around cutaneous appendages and intermuscular tissues.

No behavioral changes (e.g., scratching, aggressive behavior) or external signs of irritation (e.g., erythema, swelling, or tissue necrosis) were observed in the rats injected with the vaccine. Histological examination showed that the skin and subcutaneous tissues at the injection site remained normal, with no signs of irritation, as illustrated in Figure 10.

As illustrated in Figure 10, the study assessing the irritant effect of the QazCovid-live vaccine on the skin demonstrated no significant pathological or inflammatory reactions at the injection site. Throughout the observation period, there were no behavioral changes, such as itching, aggression, or discomfort, in the experimental group compared to the control group. Additionally, the general condition of the rats, including coat appearance and appetite, remained normal.

Histological analysis supported these findings:

• The histological structure of the skin and underlying tissues remained intact.
• Small areas of localized dermal edema were observed in both the control and experimental groups; however, these findings were clinically insignificant and unrelated to vaccine administration.
• In the experimental group, minor edema was noted around cutaneous appendages and intermuscular tissues but without any signs of inflammation, irritation, or necrosis.

These results confirm that the QazCovid-live vaccine does not induce an irritant effect on the skin or subcutaneous tissues at the injection site. The vaccine’s local tolerability is further validated by the absence of significant pathological changes observed in histological analysis.

## 4. Discussion

Depending on the stage of a pandemic outbreak, a strategy involving multiple vaccines may enhance efforts to prevent the spread and persistence of the virus within the human population. The COVID-19 pandemic has provided critical data and insights from testing diverse vaccination approaches, facilitating the development of improved strategies to address future pandemics [4,5].

Among these approaches, mRNA vaccines have demonstrated exceptional efficacy in humans and were authorized for emergency use in numerous countries during the COVID-19 pandemic. Consequently, nucleic acid-based vaccines are increasingly recognized as the foundation for next-generation vaccine development. By enabling the in vivo expression of antigenic proteins, mRNA vaccines stimulate antigen-presenting cells to process and present these antigens to CD4+ helper T cells via MHC class II pathways or to CD8+ cytotoxic T cells via MHC class I pathways. This mechanism induces robust humoral and cellular immune responses, establishing mRNA vaccines as a critical tool in pandemic preparedness [7].

Despite their high efficacy having been demonstrated in clinical trials and mass vaccination campaigns, mRNA vaccines are not without concerns, primarily regarding side effects. Among healthcare workers who received mRNA vaccines, 58.89% reported fatigue, and 21.99% experienced fever. Such side effects may contribute to vaccine hesitancy in certain populations. Additionally, the unique storage requirements of mRNA vaccines present significant logistical challenges. For instance, Moderna Inc. and BioNTech SE vaccines require storage at −30 °C and −80 °C, respectively. These conditions necessitate specialized freezers and transportation infrastructure capable of maintaining ultra-cold temperatures, which are critical for large-scale immunization programs. While this infrastructure is readily available in large, affluent cities, it poses a significant barrier in rural, sparsely populated regions and developing countries. As a result, relying solely on mRNA vaccines to combat SARS-CoV-2 globally may prove difficult, highlighting the need for diverse vaccine strategies to address these limitations [6,7].

Live attenuated vaccines expose the immune system to the entire virus, including conserved regions beyond the spike protein. This broader exposure may offer enhanced protection against variants such as Omicron, which carry mutations in the spike protein. In contrast, mRNA vaccines specifically target the spike protein, requiring compositional modifications in response to significant changes in this region. Nevertheless, mRNA vaccines are highly advantageous for rapid pandemic response due to their adaptability.

From a safety perspective, mRNA vaccines are suitable for immunocompromised individuals and pregnant women as they do not contain live virus. However, rare adverse events, such as myocarditis in children, have been reported. Live attenuated vaccines, while generally safe, are contraindicated for immunocompromised individuals due to the potential for reversion to a virulent form. Notably, live attenuated vaccines provide longer-lasting immunity, reducing the frequency of booster doses.

Given their high efficacy and short development timelines, mRNA vaccines have proven ideal for rapid pandemic response. Conversely, live attenuated vaccines are more suited for long-term immunization and population-level protection, as they mimic natural infection and induce durable immunity.

A live type of vaccine, based on a pathogen attenuated by laboratory methods or weakened in natural conditions, typically exhibits higher immunogenicity compared to others, and its production is both economical and technologically advanced [27,28,29,30,31]. Most often, such vaccines are highly effective during periods of high epidemic tension, as they are able to stimulate intense immunity in a short time, lasting for 14–21 days, as a result of a single vaccination. Whereas other types of vaccines stimulate the formation of intense immunity 7–14 days after two or more repeated uses [32,33,34,35,36].

The majority of live virus vaccines used in human and veterinary practice are derived from attenuated viruses, which are obtained by weakening their pathogenicity through multiple generations in both permissive (sensitive systems and cultivation conditions) and low-permissive (less sensitive systems and hypothermic conditions) conditions of reproduction. Vaccine virus strains obtained through these methods have proven to be reliable in terms of genetic stability, safety, and immunogenic efficacy when compared to new strains obtained through “genetic surgery”, although the durability of the specified properties and their target efficacy remain unknown. Based on this situation, the research objective of this study was to create a live dry vaccine based on the attenuated SARS-CoV-2 virus, using traditional methods and the most permissive conditions for its reproduction, with the goal of maximizing immunogenic efficacy. Multiple passages resulted in the acquisition of an apathogenic population of the SARS-CoV-2 virus, starting from the 33rd generation in the Vero cell culture, which reproduced at a temperature of 37 °C. A bioassay on susceptible animals confirmed the virus population’s lack of pathogenicity by directly introducing it into the mucous membrane of the upper respiratory tract, where infectious activity did not appear. The absence of new genetic changes over the next 67 passages confirmed the stability of the virus’s genetic system.

The attenuated multi-passage virus maintained its antigenicity, stimulating the formation of humoral immunity factors in vaccinated animals, which were detected in the neutralization reaction and serve as the primary protective elements of the body against the virulent virus.

We designate the obtained attenuated virus population as “SARS-CoV-2/RIBSP-2021” and use it as the foundation for developing a live dry vaccine against the coronavirus infection COVID-19. The Ministry of Justice of the Republic of Kazakhstan has protected the virus strain’s intellectual property with patent No. 35175.

Licensed vaccines presently depend solely on the immune response to the SARS-CoV-2 spike protein, leading to reduced efficacy and declining antibody levels over time, necessitating repeated boosters [37,38,39]. On the other hand, the entire virus has demonstrated greater robustness and longevity in eliciting immunity. Live attenuated vaccines (LAVs) have demonstrated greater efficacy than inactivated or subunit vaccines due to their ability to elicit a broader immune response. The development of the LAV in India relies on codon pair deoptimization [16]. The vaccine strain was synthetically attenuated through the incorporation of two features aimed at maximizing safety while maintaining efficacy: 1. the introduction of multiple computationally identified silent mutations in the spike gene for optimal attenuation, and 2. the deletion of 36 nucleotides encoding 12 amino acids at the furin cleavage site located between the S1 and S2 domains of the spike protein. Utilizing this codon-pair deoptimized strain, two dCoV vaccine formulations were developed: one for intranasal administration and the other for intramuscular delivery. The live attenuated vaccine serves as a suitable candidate for simultaneous immunization, offering protection against emerging SARS-CoV-2 variants [16].

Our preclinical studies provided important information on the development and safety assessment of the live-attenuated vaccine QazCovid-live. The results show that the vaccine exhibits a robust balance between safety, genetic stability, tolerability, and immunogenicity in multiple animal models. The vaccine showed no significant adverse effects, even at high doses, and elicited an appropriate immune response, making it a promising candidate for further clinical development.

By passing the SARS-CoV-2 virus through Vero cell cultures more than once, it was shown that the virus still has a consistent cytopathic effect (CPE), which is a key sign of stable viral replication. By passage 33, the virus exhibited titers ranging from 10^6.33^ to 10^7.67^ TCID_50/mL_, providing sufficient viral load for vaccine production. The stability of the CPE and the ability of the virus to elicit similar cellular responses across passage levels underscore its suitability for vaccine development.

The importance of attenuation is to reduce the pathogenicity of the virus while maintaining its ability to elicit a robust immune response [40,41]. We saw that the live QazCovid strain will cause a protective immune response without actually causing the disease it is meant to stop. This is supported by the fact that there were no significant changes in the viral titer or passage behavior.

The purification data revealed that the application of techniques like tangential flow filtration and chromatography yielded a virus yield of 5.88%, which was significantly higher than the yield from non-optimized methods. This level of yield is important for large-scale production, where efficiency is critical for meeting the needs of mass vaccination. Strict quality standards, such as low levels of endotoxins and cellular DNA, guided the purification of the virus, ensuring its safety for human use.

All experimental batches met the necessary criteria for immunobiological products, confirming the success of the purification method. Obtaining a purified virus suspension with a minimum amount of contaminants reduces the risk of adverse reactions and improves the safety profile of the vaccine.

Various experiments have optimized the freeze-drying process, which is crucial for stabilizing live attenuated vaccines [42,43]. The inclusion of lactalbumin hydrolysate and sucrose at specific concentrations provided optimal protection against viral particles during freeze-drying. The data obtained indicate that reducing the final drying period to four hours preserved the biological activity of the virus, with titer loss not exceeding 0.25–0.5 log, in contrast to the significant losses observed with longer drying times.

The ability to preserve viral biological activity after freeze-drying is critical to ensuring vaccine efficacy during storage and transportation under less than ideal conditions. This finding is particularly important for resource-limited settings where maintaining a cold chain may be challenging. A stable vaccine that retains its efficacy after reconstitution will have a significant impact on global immunization efforts [44,45].

One of the most important aspects of creating a live attenuated vaccine is to ensure the genetic stability of the attenuated virus and prevent reversion to virulence. Genetic sequencing in this study identified two stable mutations in the S gene, which remained stable across multiple passages (passages 33 to 100). The absence of additional mutations or genetic drift confirms that the attenuation process has not compromised the genetic integrity of the virus.

The S gene is responsible for encoding the spike protein, a key target for the immune response against SARS-CoV-2. The vaccine keeps these mutations so that the virus cannot go back to a pathogenic form. It also presents the spike protein in a way that makes the immune system work well. This genetic stability is critical for both safety and efficacy, as it ensures that the vaccine remains non-virulent and effective in large populations and over time [46,47,48].

We thoroughly assessed acute toxicity, one of the main markers of vaccine safety [49,50], in both mice and rats, including pregnant animals. Even at the highest doses of the vaccine, we observed no deaths in all experimental groups, making it impossible to establish an LD_50_ for the vaccine. This indicates that QazCovid-live has a wide therapeutic window, which is a very favorable property for any vaccine under development.

We measured several physiological parameters, including body weight, food and water intake, general behavior, and locomotor activity, in addition to survival. The results showed no significant deviations between the experimental and control groups, and all animals demonstrated a constant weight gain over the 14-day study period, indicating that the vaccine did not affect their metabolism or growth. These findings are further supported by the autopsy results, which showed no abnormalities in the heart, liver, lungs, kidneys, or spleen in either the experimental or control groups.

The absence of toxic effects in pregnant animals is particularly noteworthy. Given that pregnant animals are often more sensitive to toxic substances, the vaccine’s well-tolerated safety profile in this group suggests its potential applicability in pregnant women, subject to further clinical studies.

Body weight dynamics serve as a valuable indicator of general health and metabolic function, especially in toxicity studies [48,51]. In this study, there were no significant differences in weight gain between the control and experimental groups in either mice or rats, including pregnant animals. Weight gain in all groups followed a similar trend, with a constant increase recorded throughout the observation period. This uniform weight gain further underlines the non-toxicity of the vaccine.

Moreover, all animals had healthy, shiny fur and smooth skin, with no noticeable differences in vital signs such as respiratory rate, tail position, or response to visual, auditory, and tactile stimuli. These parameters are key to confirming that the vaccine did not cause systemic stress or toxicity.

We performed necropsies at the end of the study to evaluate the internal organs of the vaccinated and control animals. The liver, heart, lungs, kidneys, and other critical organs of all animals showed no pathological changes. The absence of inflammation, necrosis, or other tissue damage strongly supports the safety of QazCovid-live even at the highest doses tested.

Consistent results across species (mice and rats) and reproductive states (pregnant and non-pregnant) provide a comprehensive overview of the vaccine’s safety under various physiological conditions.

This study also provides a comprehensive subacute toxicity assessment of the QazCovid-live vaccine in laboratory rats, including male, female, and pregnant rats. Subacute toxicity assessment is critical to identify potential adverse effects upon repeated vaccine exposure [49,51]. The results of this study indicate that the QazCovid-live vaccine has a favorable safety profile, showing no evidence of significant toxicity or adverse effects even at doses ten times higher than the human therapeutic dose equivalent.

The absence of mortality in all experimental groups, including those receiving the highest doses, underlines the safety of the QazCovid-live vaccine. This result was consistent in both male and female animals, as well as in pregnant rats. Importantly, pregnant females showed no mortality or critical adverse effects, suggesting that the vaccine is likely safe for use in vulnerable groups, including pregnant rats, pending further study.

Daily observations of the experimental animals revealed no significant behavioral or physiological changes in the dose groups. Parameters such as locomotor activity, response to stimuli, coat condition, and mucous membranes remained stable, indicating that the vaccine did not cause stress or systemic toxicity. Weight gain in the experimental animals was similar to that in the control group, further supporting the conclusion that the vaccine does not adversely affect general health or metabolism. Histopathological examination of the internal organs (liver, kidneys, lungs, and heart) did not reveal significant differences between the control and experimental groups. The internal organs of the vaccinated animals appeared macroscopically healthy, with no signs of necrosis, inflammation, or other pathologies. We observed minor changes in the high dose group, such as slight edema of the liver and kidneys, but these did not indicate significant tissue damage. We observed mild proliferation of septal cells in the lungs, but this did not lead to significant changes in organ function or structure.

The vaccine had no effect on overall organ health, despite these minor observations. Organ mass ratios (MC) remained within normal physiological ranges in all groups, indicating that the vaccine did not result in organ hypertrophy or atrophy. The absence of clinical symptoms and favorable toleration to the vaccine across a wide dose range are consistent with these results.

Blood biochemistry analysis revealed a decrease in total protein and glucose levels across all groups, including the control group. These changes are attributed to the 14–15-h fasting period prior to blood sampling, reflecting a normal physiological response to nutrient deficiency and unrelated to vaccine administration. An increase in ALT levels was observed in some experimental groups; however, this change was temporary and linked to a moderate metabolic load on the liver caused by vaccine administration. Notably, other biochemical parameters, including AST, bilirubin, alkaline phosphatase, and electrolytes, remained within physiological norms, indicating no significant alterations in the functional state of the liver or other organs. Overall, these findings demonstrate good tolerability of the QazCovid-live vaccine and confirm the absence of toxic effects.

Hematological analysis revealed that most parameters, including hemoglobin levels, hematocrit, platelet counts, red and white blood cell counts, and erythrocyte sedimentation rate (ESR), were within normal physiological limits across all groups, including the control group. The increased lymphocyte percentage (80–88%) is a species-specific characteristic of the rat immune system and may also reflect a physiological response to experimental conditions, such as fasting prior to blood sampling. All other parameters remained stable, showing no significant changes, thereby confirming the absence of toxic effects associated with the administration of the QazCovid-live vaccine.

The Syrian hamster model, one of the most reliable models for studying SARS-CoV-2 due to its susceptibility to the virus, showed no clinical signs of disease or mortality after vaccination. None of the animals that received the attenuated virus showed lung pathology or signs of respiratory distress. The animals in the control group, on the other hand, lost a lot of weight and had bleeding in their lungs after being infected with the virulent strain. This showed that the wild-type virus was indeed harmful and that QazCovid-live worked to reduce its effects. These results highlight the safety of the vaccine in terms of residual pathogenicity and confirm that the attenuated virus no longer causes disease in susceptible hosts.

Safety assessments in mice have reported that the vaccine does not cause any observable pathological effects. Mice showed a sustained weight gain (0.8% to 1.0%) during the observation period, indicating that the vaccine does not interfere with normal metabolic processes. These results confirm the safety of intraperitoneal administration, adding to the growing evidence of the favorable safety profile of QazCovid-live.

In addition, safety studies in other animal models, including ferrets and kittens, did not reveal significant pathological changes in lung tissue or other organs. The study further confirmed the safety of the live attenuated strain by demonstrating that animals housed with vaccinated individuals did not transmit the attenuated virus. We detected no viral RNA or virus-neutralizing antibodies (VNA) in unvaccinated animals, confirming the absence of virus transmission from vaccinated individuals to unvaccinated contacts.

One of the main challenges with live attenuated vaccines is the potential for viral reversion to a virulent form [52,53]. For example, the attenuated virus in the oral polio vaccine can replicate within the host and, through mutation, give rise to vaccine-derived polioviruses (VDPVs). These VDPVs can reacquire neurovirulence and, in rare cases, cause vaccine-associated paralytic poliomyelitis (PAPP). Although infrequent, such adverse events are serious, and the increasing number of VDPV-related PAPP cases has garnered significant public health attention [54].

Another example is the highly effective yellow fever vaccine (strain 17D), which has been instrumental in reducing the global incidence of yellow fever. In rare instances, the attenuated virus can cause “vaccine-associated viscerotropic disease” (YEL-AVD), a condition in which the virus replicates in internal organs, mimicking severe yellow fever. Host genetic factors are thought to underlie this phenomenon. In 2001, a 56-year-old man in the United States developed multiple organ failure after yellow fever vaccination, later identified as YEL-AVD, caused by replication of the vaccine virus [55].

To mitigate the risk of pathogenic reversion, next-generation live attenuated viruses with safer and more genetically stable constructs have been developed and are now approaching practical implementation.

These considerations are particularly relevant when designing live attenuated vaccines against SARS-CoV-2. Importantly, our study demonstrated that the attenuated SARS-CoV-2 strain did not revert to a virulent form during the observation period. In three separate passages in Syrian hamsters, ferrets, and kittens, the vaccine virus was detected in the lungs of vaccinated animals, but no clinical manifestations of disease or significant pathology were observed. The lack of virus transmission and the inability of the virus to revert to a more pathogenic form further support the safety profile of QazCovid-live.

Immunogenicity studies showed that the vaccine elicited a strong immune response, particularly when administered intranasally and intratracheally. Animals vaccinated via these mucosal routes showed the highest virus-neutralizing antibody (VNA) titers, reaching 7–8 log_2_ at 21 and 28 days post-vaccination. These routes of administration may provide enhanced protection at the site of viral entry, which is critical to preventing respiratory tract infection. The intramuscular and subcutaneous routes of vaccination, on the other hand, produced lower VNA titers (3–5 log_2_ and 5–7 log_2_, respectively). This suggests that the mucosal route of vaccination may be more effective at creating a protective immune response. However, the ability to elicit a significant immune response via a variety of administration routes provides flexibility for vaccination strategies depending on population and logistical considerations.

Dose-response immunogenicity studies showed that a low dose of 1000 TCID_50_ was enough to make antibody titers that could be detected. This suggests that QazCovid-live may work at low doses. This finding is important for potential dose-sparing strategies that will allow efficient use of vaccine stockpiles in large-scale immunization campaigns.

The results showed a clear correlation between dose and the strength of the immune response. Higher doses (10^5.0^ and 10^7.0^ TCID_50_) resulted in stronger VNA titers, consistent with the principles of vaccine immunogenicity. We can administer the vaccine over a wide range of doses without causing adverse reactions, as evidenced by the absence of visible pathological effects despite increasing doses. Consistent weight gain observed in all animal groups further confirms the safety and tolerability of the vaccine.

## 5. Conclusions

The preclinical studies conducted have led to several key conclusions about the safety profile, immunogenicity, and applicability of the QazCovid-live vaccine.

Firstly, the test results confirm the high level of safety of the vaccine. All tested animals, including pregnant individuals, tolerated the vaccine well and showed no significant side effects or toxic reactions, even when administered at doses ten times higher than human doses. Observations showed no significant changes in behavior, weight, or internal organ condition, indicating the safety of the vaccine with repeated administration and at high doses. These data are important for assessing the potential use of the vaccine in various population groups, including vulnerable groups such as pregnant women.

Second, sustained production of virus-neutralizing antibodies, especially via intranasal and intratracheal administration, confirmed the vaccine’s immunogenicity and provided the best immune response. This demonstrates the potential of QazCovid-live to protect the upper respiratory tract, which is critical to combat respiratory infections such as COVID-19. At the same time, the vaccine also demonstrates flexibility in use, as it is able to induce an immune response with various routes of administration, such as subcutaneous and intramuscular, although these methods are somewhat inferior in efficiency to mucosal administration.

The third important property of the vaccine is the absence of pathogenicity and the risk of reversion to virulence. The tests confirm the stability of the attenuated virus and reduce the risk of infection during accidental contact with vaccinated animals, as the virus does not exhibit residual pathogenicity and does not transmit from vaccinated animals to contacts. This aspect is important for live attenuated vaccines, as it ensures that the virus does not revert to a virulent form, which is especially essential for a vaccine intended for mass use.

Finally, the results obtained create a solid basis for moving on to clinical trials of the vaccine in humans. The high safety profile, flexibility of dosing, and ability to induce a strong immune response make QazCovid-live a promising candidate for mass vaccination against COVID-19.

So, preclinical studies have shown that QazCovid-live has all the important qualities of a safe and effective vaccine. It is now ready for more human trials that will confirm how well it works and protects people in real life.

## Figures and Tables

**Table 1:** Results of SARS-CoV-2 virus passaging in Vero cell culture for the purpose of attenuation.

Passage	Infection Dose	Start of the Period of Manifestation of CPE, h	Cultivation Period, h	Infectious Titer of the Virus, lg TCID_50/mL_
1–5	0.01	96	125 ± 3	4.67 ± 0.28
5–10	0.01	48–72	100 ± 3	6.61 ± 0.23
10–20	0.01	36–48	96 ± 3	6.75 ± 0.21
20–30	0.01	24–36	96 ± 3	6.75 ± 0.21
30–40	0.01	24	48 ± 3	7.25 ± 0.18
40–65	0.01	24	36 ± 3	7.00 ± 0.10
66–70	0.001	24	42 ± 3	7.17 ± 0.71
71–72 c	0.0001	36	48 ± 3	6.33 ± 0.23
73–80	0.001	24	42 ± 3	7.67 ± 0.34
81–82 c	0.0001	36	48 ± 3	6.87 ± 0.18
83–90	0.001	24	42 ± 3	7.26 ± 0.54
91–92 c	0.0001	36	48 ± 3	6.69 ± 0.27
93–100	0.001	24	42 ± 3	7.34 ± 0.42
101–120	0.01	24	45 ± 3	7.67 ± 0.14

Note: c—passage viruses used for virus cloning.

**Table 2:** Virus purity indicators after purification.

Virus Suspension	Primary Treatment	Volume of CSV, L	Virus Titer, TCID_50/0.1_	Volume of PVS, L	Filter Conductivity	Purity Indicators				Virus Titer, TCID_50/0.1_	Total Virus Yield, %
						TP	ET	DNA	P		
Attenuated SARS-CoV-2 virus	Freez/Defr	18	10^7^^.^^00^	1.0	Impur.	56.9	Up to 15	22.7	1.0	10^5.50^	0.17
	Freez/Defr	31	10^6.50^	1.6	Impur.	197	Up to 15	n/r	0.6	10^5.25^	0.29
	Freez/Defr/sedim.	7	10^7.00^	1.0	Impur.	180	Up to 15	257	0.8	10^5.25^	0.25
	Freez/Defr/centrif	17	10^7.25^	1.0	Purif.	231	Up to 15	40.0	0.3	10^7.25^	5.88

Note: PVS—purified viral suspension; CSV—culture suspension of virus; TP—total protein; ET—endotoxins; DNA—cellular DNA; P—pyrogenicity.

**Table 3:** Residual virus titer in dry preparation, TCID_50/0.1 mL_.

№	Composition of the Protective Environment	Number of Components, %	Virus Titer		Drying, h	Character of Dry Tablet	Virus Titer in Dry Preparation	Virus Survival, %
			Without Stabilizer	With Stabilizer				
1	Lactalbumin Hydrolysate	8 (2.5)	10^5.5^	10^5.0^	4	Wrinkled	10^2.5^	3.1
	Sucrose	10 (4.0)						
	Sorbitol	7.5 (5.0)						
	Injection water	Up to 100						
2	Lactalbumin Hydrolysate	10 (2.5)	10^5.5^	10^5.25^	4	Compact, cylindrical	10^5.0^	58.8
	Sucrose	16 (4.0)						
	Injection water	Up to 100						
3	Lactalbumin Hydrolysate	10 (2.5)	10^7.50^	10^7.25^	4		10^6.90^	44.6
	Sucrose	16 (4.0)						
	Injection water	Up to 100						
4	Lactalbumin Hydrolysate	10 (2.5)	10^5.25^	10^4.75^	4	Compact, cylindrical	10^4.25^	30.4
	Lactose	16 (4.0)						
	Injection water	Up to 100						
5	Lactalbumin Hydrolysate	10 (2.5)	10^5.5^	10^5.0^	6	Compact, cylindrical	10^3.75^	17.0
	Sucrose	16 (4.0)						
	Injection water	Up to 100						

**Table 4:** Mass coefficients (MC) of organs in animals with intramuscular administration of the vaccine.

Indicator	Heart		Liver		Spleen		Lungs		Kidneys	
Group Number	Organ Mass, g	MC, %	Organ Mass, g	MC, %	Organ Mass, g	MC, %	Organ Mass, g	MC, %	Organ Mass, g	MC, %
№1 Control	1.30 ± 0.06	0.65	11.36 ± 0.25	5.67	1.30 ± 0.04	0.64	3.40 ± 0.14	1.70	2.41 ± 0.07	1.20
№2 Experimental	1.37 ± 0.11	0.70	10.43 ± 0.05	5.38	1.43 ± 0.07	0.73	3.36 ± 0.12	1.73	2.51 ± 0.08	1.29
№3 Experimental	1.50 ± 0.10	0.75	10.89 ± 0.22	5.51	1.35 ± 0.09	0.68	2.48 ± 0.15	1.25	2.46 ± 0.03	1.24
№4 Experimental	1.40 ± 0.14	0.72	11.43 ± 0.41	5.85	1.37 ± 0.04	0.70	2.86 ± 0.43	1.46	2.48 ± 0.17	1.27
№5 Pregnant (Control)	1.44 ± 0.05	0.56	10.92 ± 0.20	4.28	1.51 ± 0.05	0.59	3.39 ± 0.24	1.33	2.61 ± 0.12	1.02
№6 Pregnant (oпытнaя)	1.54 ± 0.20	0.62	11.43 ± 0.41	4.65	1.37 ± 0.04	0.55	2.86 ± 0.43	1.16	2.48 ± 0.17	1.01

**Table 5:** Biochemical parameters of animal blood (rats) in the study of subacute toxicity.

Blood Biochemistry Test Indicators	Test System Group Number						
	Standard	№ 1	№ 2	№ 3	№ 4	№ 5	№ 6
Bilirubin (mmol/L)	0.0–1.67	1.13 ± 0.17	1.14 ± 0.13	1.14 ± 0.13	1.23 ± 0.11	0.62 ± 0.25	1.25 ± 0.07
Alkaline phosphatase (U/L)	160–960	165.10 ± 14.58	280.72 ± 5.54	372.04± 15.00	367.12± 7.66	172.10 ± 8.51	170.55 ± 1.34
Creatinine (mol/L)	68–104	94.85 ± 9.88	87.12 ± 11.82	76.04 ± 4.57	63.86 ± 6.30	87.20 ± 1.85	92.25 ± 3.04
Total protein (g/L)	98–108	79.05 ± 1.94	80.90 ± 2.73	81.24 ± 1.93	83.14 ± 3.79	91.17 ± 4.48	87.25 ± 0.49
Uric acid (mmol/L)	8–14	7.20 ± 0.53	6.36 ± 1.02	7.44 ± 1.11	7.94 ± 2.72	7.73 ± 3.78	8.20 ± 0.14
AST (IU/L)	150–300	181.93 ± 11.55	225.46 ± 7.12	226.34 ± 13.11	275.40 ± 6.71	178.93 ± 6.13	194.80 ± 2.26
ALT (IU/L)	110–140	133.98 ± 11.69	161.68 ± 8.36	157.98 ± 7.54	219.88 ± 14.10	159.23 ± 7.80	198.10 ± 3.54
Glucose (mmol/L)	50–135	6.52 ± 1.26	6.34 ± 0.78	6.54 ± 0.43	6.64 ± 0.42	6.33 ± 0.68	6.15 ± 0.07

**Table 6:** Hematological indices of the general blood analysis of animals (rats) in the experiment subacute toxicity.

Hemogram Test Results	Test System Group Number						
	Standard	№ 1	№ 2	№ 3	№ 4	№ 5	№ 6
Leukocytes (10^9^/L)	5–23	6.16 ± 0.23	13.04 ± 4.12	12.20 ± 3.20	8.30 ± 2.01	6.50 ± 1.47	5.25 ± 0.21
Lymphocytes (%)	50–70	81.72 ± 7.94	85.82 ± 2.75	80.32 ± 6.31	84.02 ± 3.11	88.10 ± 3.86	86.40 ± 0.42
Hemoglobin (g/L)	120–180	150.60 ± 12.92	150.60 ± 12.92	154.60 ± 2.70	161.80 ± 12.50	161.67 ± 14.01	165.00 ± 11.30
Hematocrit (%)	35–52	49.66 ± 3.86	46.60 ± 3.91	48.48 ± 0.86	52.64 ± 5.74	51.50 ± 7.50	47.85 ± 3.04
Platelets (10^9^/L)	200–600	444.60 ± 11.30	362.40 ± 18.80	522.20 ± 15.64	384.20 ± 13.59	363.33 ± 6.03	400.00 ± 2.83
Erythrocytes (10^12^/L)	7–10	9.23 ± 0.59	8.64 ± 0.71	9.22 ± 0.24	9.37 ± 0.52	8.97 ± 1.06	9.03 ± 0.07
Neutrophils (%)	2–30	3.46 ± 1.09	3.72 ± 0.93	4.28 ± 1.84	2.98 ± 0.59	3.27 ± 0.15	3.20 ± 0.14
ESR (mm/h)	1–3	1.40 ± 0.55	1.20 ± 0.45	2.20 ± 1.79	1.20 ± 0.45	1.33 ± 0.58	1.33 ± 1.53

**Table 7:** Pathogenicity of the 80th and 100th passage virus with different methods of administration.

Animals	Number of Animals	Virus, Passage Level	Virus Dose, TCD_50_	Methods of Administration	Clinical Disease	Presence of Pathologies in the Lungs
Syrian hamsters, intact	5 + 5	80th and 100th	10^4.5^	s/c	0/0	0
	5 + 5		10^4.5^	i/v	0/0	0
	5 + 5		10^4.5^	i/n	0/0	0
	5 + 5		10^4.5^	i/t	0/0	0
	10	5	10^4.5^	i/n	2/10	10

Note: the denominator is the number of sick animals, the numerator is the number of dead animals; s/c—subcutaneous; i/v—intravenous; i/n—intranasal; i/t—intratracheal.

**Table 8:** Results of passaging the attenuated virus in Syrian hamsters, ferrets, and kittens by intranasal inoculation at a dose of 10^6.0^ TCID_50/head_ to assess reversibility and possible transmission to intact animals.

Virus Passages	Animals, Species	Number of Animals, Head	Timeframe for Planned Forced Slaughter After Vaccination, Days			Got Sick	Survived	Animals with Development of Pathologies in the Lungs	Daily Live Weight Gain, g	Presence of Virus or RNA in the Lungs	Presence of VNA in Blood Serum
			3	5	7						
First	Syrian Hamsters	10	2	2	2	0	4	0	0.9–1.3	6/6	4/4
		10 *	-	-	-	0	10	0	1.1–1.5	0/10	0/10
	Kittens	6	1	1	1	0	3	0	n/t	3/3	3/3
	Ferrets	5	1	1	1	0	2	0	n/t	3/3	2/2
Second	Syrian Hamsters	12	2	2	2	0	6	0	1.1–1.4	6/6	4/4
	Kittens	4	1	1	n/t	0	2	0	n/t	2/2	0/2
	Ferrets	5	1	1	1	0	2	0	n/t	2/2	1/2
Third	Syrian Hamsters	12	2	2	2	0	6	0	0.8–1.4	2/6	0/6
	Kittens	4	1	1	n/t	0	2	0	n/t	0/2	0/2
	Ferrets	5	1	1	1	0	2	0	n/t	1/3	0/2
-											

Note: *—animals kept together with those vaccinated with an attenuated virus, n/t—not tested; - the denominator shows the number of tested animals, the numerator shows the number of positive animals.

**Table 9:** EI (edema index) values of experimental animals in DTH studies.

№1 Experimental Group				№4 Control Group		
№	M_o_	M_к_	EI	M_o_	M_к_	EI
1	0.1175	0.1082	8.59	0.1492	0.1365	9.31
2	0.1285	0.1196	7.44	0.1435	0.1326	8.22
3	0.1571	0.1435	9.47	0.1446	0.1374	5.24
4	0.1288	0.1191	8.14	0.1278	0.1158	10.36
5	0.1481	0.1373	7.86	0.1371	0.1311	4.57
6	0.1392	0.1334	4.34	0.1481	0.1361	8.82
7	0.1491	0.1377	8.27	0.1205	0.1128	6.83
8	0.1565	0.1485	5.38	0.1253	0.1191	5.21
9	0.1391	0.1322	5.22	0.1517	0.1431	6.01
10	0.1501	0.1383	8.53	0.1472	0.1392	5.75
			7.32 ± 1.63			7.03 ± 1.90
**№2 Experimental Group**				**№5 Control Group**		
**№**	**M_o_**	**M_к_**	**EI**	**M_o_**	**M_к_**	**EI**
1	0.1615	0.1521	6.18	0.1711	0.1613	6.07
2	0.1539	0.1413	8.92	0.1491	0.1383	7.81
3	0.1321	0.1222	8.10	0.1638	0.1541	6.29
4	0.1261	0.1172	7.59	0.1712	0.1605	6.67
5	0.1227	0.1128	8.77	0.1636	0.1524	7.35
6	0.1491	0.1365	9.23	0.1538	0.1449	6.14
7	0.1675	0.1555	7.72	0.1594	0.1478	7.85
8	0.1621	0.1562	3.78	0.1821	0.1719	5.93
9	0.1571	0.1497	4.94	0.1862	0.1749	6.46
10	0.1711	0.1625	5.29	0.1643	0.1498	9.68
			7.05 ± 1.79			7.03 ± 1.11
**№3 Experimental Group**				**№6 Control Group**		
**№**	**M_o_**	**M_к_**	**EI**	**M_o_**	**M_к_**	**EI**
1	0.1382	0.1266	9.16	0.1487	0.1374	8.22
2	0.1643	0.1563	5.12	0.1478	0.1426	3.65
3	0.1319	0.1237	6.63	0.1465	0.139	5.39
4	0.1386	0.1268	9.31	0.1478	0.1351	9.40
5	0.1524	0.1447	5.32	0.1761	0.1631	7.97
6	0.1323	0.1227	7.82	0.1508	0.1441	4.65
7	0.1551	0.1468	5.65	0.1563	0.1451	7.72
8	0.1483	0.1395	6.31	0.1638	0.1506	8.76
9	0.1638	0.1518	7.91	0.1571	0.1432	9.71
10	0.1443	0.1329	8.58	0.1456	0.1375	5.89
			7.18 ± 1.49			7.14 ± 1.98

**Table 10:** Skin reaction results.

Reaction (Score)	Evaluation Scale	
	Control	Experimental Group
Pale pink erythema throughout the area or at its periphery	_	_
Bright pink erythema over the entire area or its periphery	_	_
Red erythema over the entire area	_	_
Infiltration and edema of the skin (thickening of the skin fold) with or without erythema	_	_
Erythema, severe infiltration, focal ulcerations (necrosis), hemorrhages, crusting are possible	_	_

## Data Availability

Data is contained within the article.
